# Diphtheria*Read before the Nebraska State Medical Society at Omaha, May, 1892.

**Published:** 1892-08

**Authors:** A. Bowen

**Affiliations:** Nebraska City, Neb.


### DIPHTHERIA.*

BY A. BOWEN, M. D., NEBRASKA CITY, NEB.

Eleven years since, at the request of this Society, I addressed you upon this same subject, and if to-day at the request of your chairman of Committee on Practice of Medicine, I come before you and say that I have no new suggestions to make on treatment, you may think that I lack the spirit of progressiveness; and that the light which science sheds over all things, had not visited my eyes.

The revelations of nature are old and the power of drugs remain the same to us that they did to our fathers.

---

*Read before the Nebraska State Medical Society at Omaha, May, 1892.

The same treatment which I used to abort tonsillitis forty years since, in the Catskill mountains, is all-sufficient, and has always been, in extinguishing diphtheria. If the patients are seen early and treated from the first with *overbearing and persistent earnestness,* nothing less than this will answer, and this always will. I am not blind to the fact that different epidemics of diphtheria, like different epidemics of small-pox and scarlatina, possess different degrees of malignity, and will claim their victims. But I do know, that all of you who are *terribly in earnest* can limit the mortality to less than five per cent. I do know (*I do not conjecture*) that German children are more obnoxious to the disease than American children. Probably this is due to their different mode of living.

Three years since, I was called to two families of Germans eighteen miles from my residence. Seven members, who were children, in one family, and four in the other. They had contracted the disease from the contagion of a corpse at a funeral. One in each family had the disease for several days before I was called. I said to the parents that those two would die. Knowing that my word was law in those two houses, I unhesitatingly commenced the same course of treatment through the day, among the sick and the well. Among them was a baby who nursed a bottle. Their throats were earnestly swabbed every two hours during the day, and the sick during the day and the night.

As fast as they came down with the disease, I swabbed during the night as well as during the day. Every child had the disease, and every child except the one in each family, recovered. These two were the last children I lost of diphtheria. When the epidemic commenced last November, the chairman of the Board of Education wrote to all the medical men in town, requesting their opinion of the necessity of closing the public schools. All but one dissuaded them from closing. It was the mildest and the widest spread epidemic of diphtheria I ever saw. Still, there is enough diphtheria in every case to kill, if neglected. I always grow poor in flesh when treating diphtheria. As a gargle for children who are exposed to contagion or infection, a 4 per cent. solution of carbolic acid, thoroughly mixed with cold strained tea, either gargled, or better yet, mopped deep in the throat, every four hours, or if the disease has invaded the nostrils or nares, or threatens to, the same wash sent through a two or three drachm glass syringe, with somewhat forcible jet, a small cork being fitted to the nostrils of the child with an aperture through the cork to fit the nozzle of the syringe; and the child always held perpendicular. If the disease has invaded the throat, as it usually has then, more earnest means must be used there.

Of twelve parts by measure:

One part must be carbolic acid; three parts sulphurous acid; four parts muriatic tincture iron; four parts glycerine.

The throat to be swabbed or mopped deep with this solution every two, three or four hours. They must eat and they must sleep, and here comes in the tact and discretion of a good nurse and all you possess of these qualities, you will find available. Intubation is a more respectable apology than laryngotomy, but the latter is a merely bloody excuse for early neglect. *Arrest* the disease at its earliest moment, and save the patient from its terrible alternatives, asphyxia or septicæmia.

If your patient resists you, swathe him or her tightly from the chin to the toes, and get the mouth open by the help of a soft pine wedge. They will not resist you if their throats have been properly educated.

As to the cause of diphtheria, we do not possess much more accurate information than of the cause of small-pox, or scarlatina, or the epizootic. We hear that the latter disease has struck our Eastern coast, and we know that in about so many days it will show itself in our stables, though the wind may blow a gale from the west all the intervening time.

"Diphtheria is surely a pestilence that walketh in darkness." Belknap says, in his history of New Hampshire, that a visitation of what was probably, from the description, diphtheria, first visited our country in 1735. Of the first forty who were attacked, none recovered. In the parish of Hampton Falls, twenty-seven persons died in five families, and one-sixth of the population died in thirteen months, and twenty families buried all their children. We may have reason to fear (for we have seen a similar error committed in our day) that some physicians may have depleted for the wretched fever which accompanies the disease, forgetting that it is adynamic in its character.

Belknap says of the Boston visitation at this period: "The physicians having, by desire of the select men, held a consultation, published their opinion that it proceeded entirely from *some occult quality in the air.*" Can their successors of the fourth generation give a more definite answer with assurance? And at what earlier day did an American town institute *scientific inquiries* as the basis of sanitary measures?

I cannot look upon diphtheria even in our present ignorance of its prime cause, as one of the *opprobria medicorum*, but as a disease to be met earnestly, and in a hopeful mood.

